# Molecular detection of *Theileria* species and *Babesia caballi* from horses in Nigeria

**DOI:** 10.1007/s00436-020-06797-y

**Published:** 2020-07-10

**Authors:** Philip W. Mshelia, Lowell Kappmeyer, Wendell C. Johnson, Caleb A. Kudi, Okubanjo O. Oluyinka, Emmanuel O. Balogun, Edeh E. Richard, Emmanuel Onoja, Kelly P. Sears, Massaro W. Ueti

**Affiliations:** 1 Program in Vector-borne Diseases, Department of Veterinary Microbiology and Pathology, Washington State University, Pullman, WA 99164-7040 USA; 2 Department of Veterinary Medicine, Ahmadu Bello University, Zaria, Kaduna 810107 Nigeria; 3 Animal Diseases Research Unit, USDA-ARS, Pullman, WA 99164-6630 USA; 4 Department of Veterinary Parasitology and Entomology, Ahmadu Bello University, Zaria, Kaduna Nigeria; 5 Department of Biochemistry, Ahmadu Bello University, Zaria, Kaduna 810107 Nigeria; 6 Department of Veterinary Medicine, Surgery and Radiology, University of Jos, Jos, Plateau State Nigeria; 7 The Paul G. Allen School for Global Animal Health, Washington State University, Pullman, WA 99164-70403 USA

**Keywords:** *Theileria equi*, *Babesia caballi*, Horses, Equine piroplasmosis

## Abstract

Equine piroplasmosis (EP) is an infectious, tick-borne disease caused by the hemoprotozoan parasites, *Theileria equi*, *Babesia caballi*, and a recently reported new species, *T. haneyi*. Infections by these apicomplexan parasites limit performance and cause economic losses for the horse industry. Equine piroplasmosis is widespread in the northern regions of Nigeria, where an increasing portion of the animal population is composed of horses. This disease has remained epidemiologically challenging, especially as the movement of horses increases across Nigeria. In this study, blood samples from 300 horses were collected in three states of northwestern Nigeria. The presence of piroplasms was screened by nested PCR targeting 18S rDNA and positive samples were analyzed using species-specific-nested PCR-targeting genes including *ema1* (*T. equi*), *rap1* (*B. caballi*), and a gene coding a protein of unknown function (*T. haneyi*). Species-specific-nPCR results demonstrated that the prevalence of *T. equi* was 13.0% (39/300), *B. caballi* was 3.3% (10/300) and *T. haneyi* was 2.7% (8/300). Mixed infections with *T. equi* and *B. caballi* was 2.7% (8/300) while *T. equi*, *B. caballi*, and *T. haneyi* multiple infection prevalence was 0.6% (2/300). We used 18S rDNA sequences to determine close relationships between *T. equi* by phylogenetic analysis and demonstrated that among 57 sequences of *Theileria* parasites, 28 samples belonged to clade A (49%), 13 samples were found to be clade C (22%), and 16 were clade D (28%). These results demonstrate the genetic diversity of *T. equi* circulating in horses from Nigeria.

## Introduction

Equine piroplasmosis (EP) is an infectious, tick-borne disease caused by the hemoprotozoan parasites *Theileria equi*, *Babesia caballi*, and a recently reported new species, *T. haneyi* (Hall et al. 2013; Scoles and Ueti 2015; Knowles et al. 2018). It has a global distribution, but a few countries are reported to be EP-free (Wise et al. 2013; Scoles and Ueti 2015; World Organisation for Animal Health 2019). Genera of Ixodid ticks, namely *Rhipicephalus*, *Dermacentor*, and *Hyalomma*, are known to transmit these protozoa (Scoles and Ueti 2015). These protozoan pathogens cause acute and chronic infections with a mortality rate of up to 50% (de Waal 1992). Horses infected with *T. equi* remain persistently infected while those affected by *B. caballi* are infected for an extended period before the immune system naturally eliminates the parasite without therapeutic intervention. Conversely, the parasitemia may fall below the level of detection (Friedhoff and Soule 1996). Chronically infected horses are reservoirs to spread the pathogens via tick or iatrogenic transmission (Ueti et al. 2008; Short et al. 2012). It was reported that poor management of equine populations might have exacerbated the impact of infection in the most endemic areas (Scoles and Ueti 2015). Timoney (2000) suggests that the international trade in horses and other equids could lead to an increase in the global spread of equine diseases.

Previous reports have suggested that global warming has resulted in changes in ecosystems as well as microbial ecology (Harvell et al. 2002). The recent EP outbreaks in the USA and the Netherlands, without visible premonitory signs and reduced detectability during long-term persistence (Short et al. 2012; Butler et al. 2012), have made EP a challenging disease. The discovery of *T. haneyi*, defined as a new species infective to equids that lacks *ema-1*, the current diagnostic test target, allows the new parasite to elude current regulatory tests (Knowles et al. 2018). To overcome this issue, a gene coding a protein of unknown function in the syntenic *T. haneyi* locus of the vacated *ema1* gene was selected to detect *T. haneyi* by PCR assay (Knowles et al. 2018; Sears et al. 2019). *T. haneyi* was initially thought to belong to a new isolate of *T. equi*, but phylogenetic evidence supports that it diverged from *T. equi* (Knowles et al. 2018). Previous studies based on phylogenetic analysis showed genetic diversity among *T. equi* isolates across the world, including clades A, B, C, D, and E, with the recently identified novel species *T. haneyi* belonging to clade C (Knowles et al. 2018).

Horses from various regions in northern Nigeria infected with protozoa parasites that cause EP were reported without substantive information on molecular detection and genetic diversity (Oladosu and Olufemi 1992). The mechanisms of EP transmission in Nigeria are poorly characterized. It is unknown which tick species in Nigeria are competent vectors for the pathogens that cause EP. Several ticks have been implicated in transmission of EP to horses, including *Rhipicephalus evertsi evertsi*, *Rhipicephalus decoloratus*, *Hyalomma impeltatum*, *Hyalomma. truncantum*, *Hyalomma dromederii*, and *Amblyomma variegatum* (Onyiche et al. 2019; Scoles and Ueti 2015), and some of these are found in Nigeria (Oguntomole et al. 2018), though not on equids. However, horses are not the preferred host for some ticks identified, and their natural disease relationships are not well-known. Besides tick transmission, other cogent factors contribute to the mechanical of EP transmission. These include injections with contaminated needles and syringes, bloodletting, and “acupuncture-like” practices (Mshelia PW, personal observation).

The diagnosis of EP has been based mostly on microscopic examination of blood smears and serological assays (Oladosu and Olufemi 1992; Knowles Jr. et al. 1992). However, the limitations of these diagnostic tools made it difficult to identify and genetically characterize species of *Babesia* and *Theileria* present in Nigeria. Horses in Nigeria are used for leisure riding, polo games, horse racing, and traditional ceremonies resulting in extensive horse movement across Nigeria without strict control. This could impact the epidemiology of EP. Therefore, in this study, emphasis was given to the molecular detection of these parasites, the causative agents of equine piroplasmosis, to begin to define their importance in Nigeria.

## Materials and methods

### Sample collection from horses

**Fig. 1:**
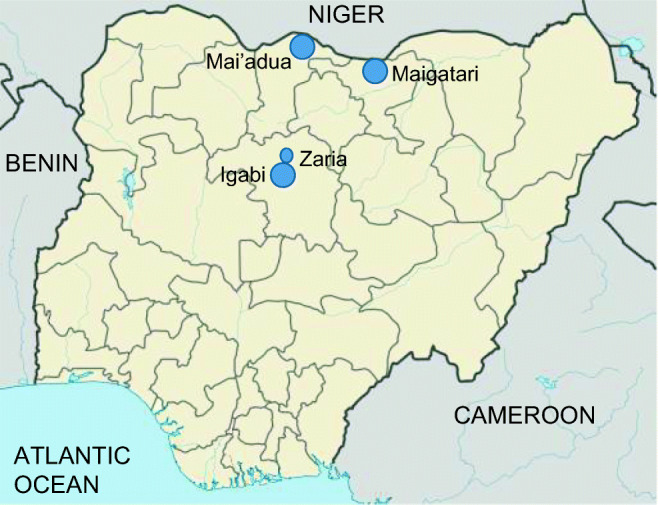
Geographic location of the study sites in Nigeria. The map was modified from Wikipedia

All procedures performed in this study involving animals were in strict compliance with the ethical standards of Ahmadu Bello University, Zaria, Nigeria, ABUCAUC/2020/34. Blood samples were collected from 300 horses ranging from 2.5 to 18 years old from three States in northwestern Nigeria (Fig. 1), namely Jigawa (Maigatari: 12° 48′ 18.15′′ N, 9° 26′ 52.13″ E) Kaduna (Zaria 11° 2′ 57.18′′ N, 7° 41′ 56.39″ E; Igabi 10° 48′ 23.53′′ N, 7° 42′ 55.82″ E), and Katsina (Mai’adua 13° 10′ 45.00′′ N, 8° 13′ 48.98″ E). Samples from horses at livestock border markets (Maigatari, Kaduna state; Mai’adua, Katsina state) and samples from 8 horses imported from Argentina (Polo pony) were collected to determine if imported horses are infected with pathogens that cause equine piroplasmosis. Samples from resident horses, including polo horses with permission by owners, were selected to determine the presence of equine piroplasmosis (Igabi, Kaduna state, polo ponies; Zaria, Kaduna state; polo ponies and Dubar horse procession). Table 1 describes samples collected from horses in Nigeria.

**Table 1:** Description of horse samples collected in Nigeria

Area	Number of horses	Breed	Use
Kaduna			
• Igabi	80	Argentine polo ponies	Polo
	8	Thoroughbred	Polo
	3	Criollo	Leisure
• Zaria	127	West African Barb	Leisure
	25	West African Barb	Race
Katsina			
• Mai’adua	32	West African Barb	Leisure
Jigawa			
• Maigatari	25	West African Barb	Leisure

Samples were collected in a vacutainer from the external jugular vein of randomly chosen animals and transported to the Department of Veterinary Medicine, Faculty of Veterinary Medicine, Ahmadu Bello University, Zaria, Nigeria. One hundred fifty microliters of blood was applied to Whatman FTA cards (GE Healthcare companies, USA). The application began at the center of the circle and moved out spirally towards the edge to result in a uniform distribution and the opportunity to isolate DNA from repeated sample punches. After applying the blood, the card was stored at room temperature for 6 h to dry. The dried card was sealed in a re-sealable bag with a pack of desiccant and stored in a cool, dry, and dark place until analysis.

### DNA extraction

A sample disc approximately 4 mm in diameter was taken from each sample spot using a disposable biopsy punch with a plunger (Integra Life Sciences Services, France). The sample discs were placed in 0.2-mL PCR tubes. Two hundred microliters of FTA Purification Reagent (Whatman TM GE Healthcare UK Limited, UK) was added to each PCR tube and incubated for 5 min at room temperature. The supernatant was discarded. This procedure was repeated for a total of 3 washes with FTA Purification Reagent. The discs were suspended in 200 μl of TE buffer (10 mM Tris-HCl, 0.1 mM EDTA, pH 8.0) and incubated for 5 min at room temperature. The same procedure was repeated for a total of 2 washes with TE buffer. The discs were then allowed to dry at room temperature for 1 h. The FTA disc was used for PCR amplification.

### Nested PCR amplification of the V4 region of 18S rRNA gene

A hemi-nested PCR (the reverse primer being used in each reaction) assay was used to amplify the hypervariable region of the 18S rRNA gene as previously described (Liu et al. 2016). Briefly, the external PCR reaction was set up in a 19-μl total volume containing 10 μl Sigma 2× Jumpstart Red Taq mix, 7 μl of nuclease-free water, 1 μl of 10 μM RLB-F2, and RLB-R2 primers (Table 2) and the processed DNA disc from the FTA card. The external PCR reaction consisted of an initial denaturation at 95 °C for 3 min, followed by 35 cycles of 95 °C for 60 s, 52 °C for 50 s, and 72 °C for 90 s. The final extension was performed with one step at 72 °C for 5 min. The internal PCR reaction was set up in a 20-μl total volume containing 10 μl Sigma 2× Jumpstart Red Taq mix, 7.5 μl of nuclease-free water, 1 μl of 10 μM RLB-FINT, and RLB-R2 primers and 0.5 μl of the first PCR product. The reaction cycling in the second PCR was carried out with an initial denaturation at 95 °C for 3 min, followed by 35 cycles of 95 °C for 30 s, 50 °C for 30 s, and 72 °C for 30 s, and a final extension was performed at 72 °C for 5 min. PCR products were electrophoresed on a 1.5% agarose gel containing SYBR Safe DNA gel stain (Invitrogen, USA) in Tris-acetate-EDTA (TAE) buffer at 100 V for 35 min and visualized under UV light.

**Table 2:** Primer sets used to determine horses infected with protozoan parasite in Nigeria

Species	Primer	Size (bp)	Sequence 5′-3’	Tm (°C)	Reference
18 s	RLB-F2	399	GACACAGGGAGGTAGTGACAAG	52	Liu et al. (2016)
18 s	RLB-FINT	383	GACAAGAAATAACAATACRGGGC	50	
18 s	RLB-R2	399	CTAAGAATTTCACCTCTGACAGT	52	
*B. caballi*	Bc_extfor	229	GATTACTTGTCGGCTGTGTCT	60	Schwint (2008)
*B. caballi*	Bc_intfor	221	GCTAAGTACCAACCGCTGA	60	
*B. caballi*	Bc_rev	221	CGCAAAGTTCTCAATGTCAG	60	
*T. equi*	Te_extfor	229	GAGGAGGAGAAACCCAAG	60	Baptista et al. (2013)
*T. equi*	Te_extrev	229	GCCATCGCCCTTGTAGAG	60	
*T. equi*	Te_intfor	229	TCAAGGACAACAAGCCATAC	60	
*T. equi*	Te_intrev	229	TTGCCTGGAGCCTTGAAG	60	
*T. haneyi*	Th_extfor	238	CCATACAACCCACTAGAG	63.5	Knowles et al. (2018)
*T. haneyi*	Th_extrev	238	CTGTCATTTGGGTTTGATAG	63.5	
*T. haneyi*	Th_intfor	238	GACAACAGAGAGGTGATT	58.1	
*T. haneyi*	Th_intrev	238	CGTTGAATGTAATGGGAAC	58.1	
*B. bovis*	Bb_extfor	360	CACGAGGAAGGAACTACCGATGTTGA	60	Figueroa et al. (1993)
*B. bovis*	Bb_extrev	360	CCAAGGAGCTTCAACGTACGAGGTCA	60	
*B. bovis*	Bb_intfor	291	TCAACAAGGTACTCTATATGGCTACC	60	
*B. bovis*	Bb_intrev	291	CTACCGAGCAGAACCTTCTTCACCAT	60	

### Nested PCR targeting species-specific parasites

All 18S-nested PCR positive samples were analyzed using nPCR (hemi-nested for *B. caballi rap1*) to identify specific piroplasms present in the samples. Primer sets for *T. equi* (*ema-1* gene), *T. haneyi* (gene coding protein of unknown function), and *B. caballi* (*rap-*1 gene) are shown in Table 2. The reaction conditions for *T. equi* and *B. caballi* nPCR are as follows: the PCR external reaction was prepared with 12.5 μl 2× Platinum SuperFi PCR master mix (Invitrogen, USA), 1 μl of 10 μM of each primer, the FTA disc, and 9.5 μl of nuclease-free water. The PCR cycling conditions for *T. equi* and *B. caballi* were a denaturation at 98 °C for 5 min, followed by 35 cycles of 98 °C for 20 s, 59.5 °C for 20 s, and 72 °C for 20 s, with a final extension at 72 °C for 5 min. In the internal PCR reaction, 1.0 μl of each PCR product from the external PCR reaction was used as a template and added to 12.5 uL 2× Platinum Superfi master mix and 11.5 uL of water. The internal reaction consisted of denaturation at 98 °C for 5 min followed by 35 cycles of 98 °C for 5 s, 61 °C for 5 s, and 72 °C for 5 s, with a final extension at 72 °C for 5 min (Baptista et al. 2013; Schwint et al. 2009).

For *T. haneyi*, the PCR external reaction was prepared with 12.5 μl 2× Platinum SuperFi PCR master mix (Invitrogen, USA), 1 μl of 10 μM of each primer, the FTA disc, and 9.5 μl of nuclease-free water. The cycling condition for the external reaction of *T. haneyi* included an initial denaturation at 95 °C for 4 min., followed by 35 cycles of 95 °C for 20 s, 63.5 °C for 30 s, and 72 °C for 20 s and a final extension at 72 °C for 7 min (Knowles et al. 2018; Sears et al. 2019). The PCR internal reaction was prepared in a final volume of 25 μl, with 12.5 μl 2× Platinum SuperFi PCR master mix (Invitrogen, USA), 1 μl of 10 μM of each set of primers, 1 μl of DNA template from the external reaction, and 9.5 μl of nuclease-free water. The external reaction cycle includes initial denaturation at 95 °C for 4 min., followed by 35 cycles of 95 °C for 20 s, 58.1 °C for 30 s, and 72 °C for 20 s and a final extension at 72 °C for 7 min.

Finally, a *Babesia bovis* nPCR based on the *rap-1* gene was applied to two equine DNA samples (Thatcher198 and Castora213 RC) for which 18S-PCR and sequencing unexpectedly resulted in *B. bovis* 18S rDNA identity. The nPCR primers targeting *B. bovis rap-1* gene are reported in Table 2, and performed according to the procedure reported elsewhere (Figueroa et al. 1993).

### Phylogenetic analysis

The 18S rDNA nPCR products from samples that were positive for 18S nPCR but negative for a species-specific reaction were sequenced to identify other parasites infecting horses. The samples were amplified for the V4 region of the 18S rRNA gene. The PCR products were cleaned up using ExoSAP-IT reagent (Applied Biosystems, Lithuania) before being sent for sequencing using amplifying primers (Sanger sequencing method, Eurofins Genomics, SimpleSeq service, Louisville, KY, USA). Sequencher (Genecodes, Ann Arbor, MI, USA) was used to trim raw sequences, and the resulting sequences were analyzed at the NCBI website (https://blast.ncbi.nlm.nih.gov/Blast.cgi) using nucleotide Blast with default settings. Reference sequences for 18S rDNA representing *T. equi* genotypes A–E, *T. haneyi*, *B. caballi*, and *B. bovis* were retrieved from GenBank for inclusion in the phylogenetic analysis (Bhoora et al. 2009; Knowles et al. 2018; Hall et al. 2013). The reference sequences are for *T. equi* clade A (JX177671, Z15105), *T. equi* clade B (AB515310), *T. equi* clade C (JQ390047, EU888903), *T. equi* clade D (AB515307, AB515312), and *T. equi* clade E (HM229408). Reference sequences for *B. bovis* (AY150059) and *B. caballi* (EU642512) were also included in the analysis as outgroups.

### Evolutionary analysis by maximum likelihood method

**Fig. 2:**
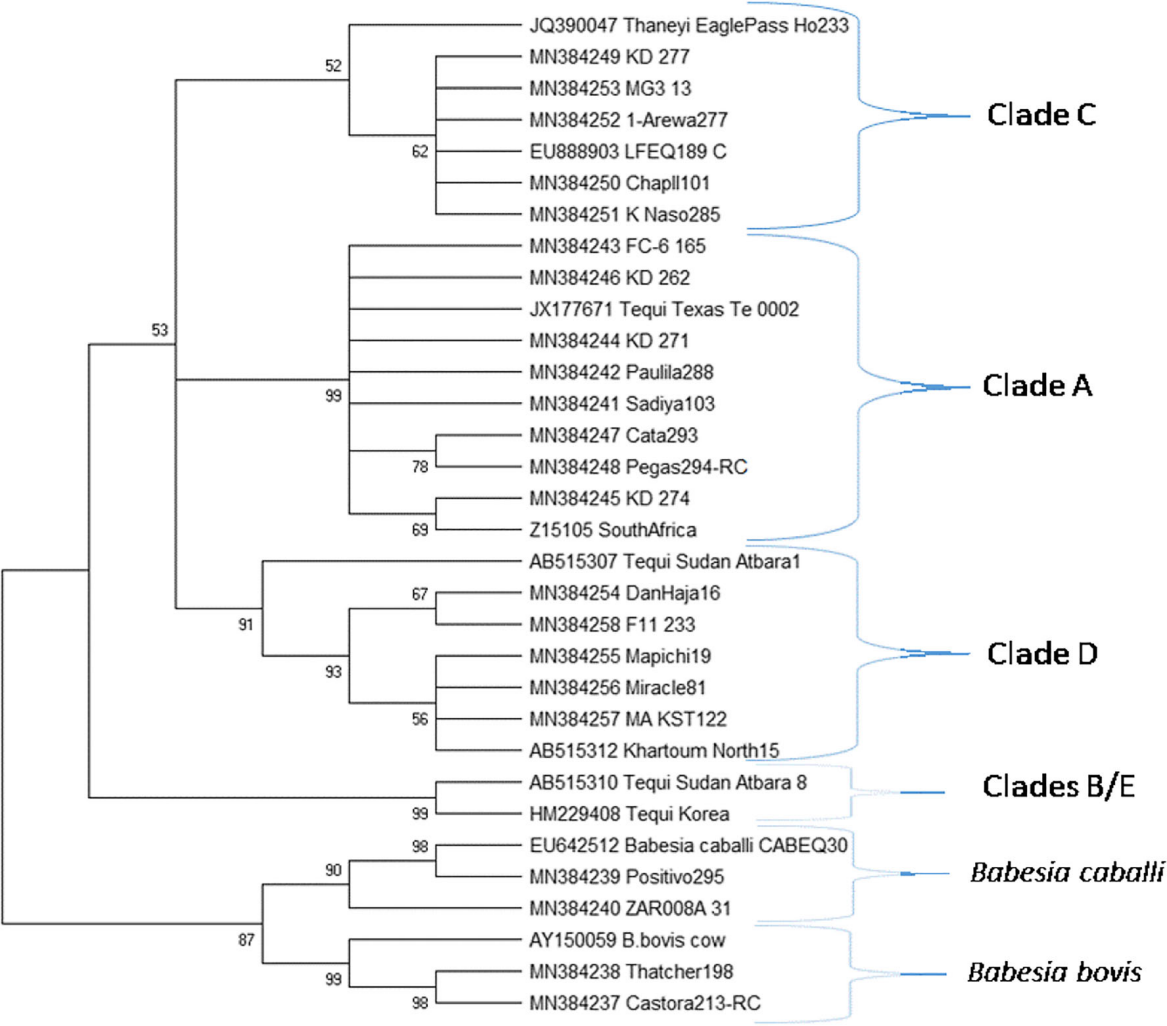
18S rDNA maximum likelihood analysis of northwestern Nigerian equine samples with comparison to hallmark cases. All samples with accession numbers beginning with MN are new, generated from this work. The phylogram is based on sequence variation covering the V4 region of the 18S rDNA gene

The evolutionary history was inferred by using the maximum likelihood method and Kimura 2-parameter model (Kimura 1980). The bootstrap consensus tree inferred from 1000 replicates is taken to represent the evolutionary history of the taxa analyzed (Felsenstein 1985). Branches corresponding to partitions reproduced in less than 50% bootstrap replicates are collapsed. The percentage of replicate trees in which the associated taxa clustered together in the bootstrap test (1000 replicates) is shown next to the branches (Felsenstein 1985). Initial tree(s) for the heuristic search were obtained by applying the neighbor-joining method to a matrix of pairwise distances estimated using the maximum composite likelihood (MCL) approach. There was a total of 319 positions in the final dataset and a tree is presented in Fig. 2. Evolutionary analyses were conducted in MEGA 6 (Kumar et al. 2018) and 22 randomly selected sequences from distinct areas were used for phylogenetic analysis. NCBI accession numbers were obtained for the 22 generated Nigerian 18S rDNAsequences used in the phylogenetic analysis (MN384237, MN384238, MN384239, MN384240, MN384241, MN384242, MN384243, MN384244, MN384245, MN384246, MN384247, MN384248, MN384249, MN384250, MN384251, MN384252, MN384253, MN384254, MN384255, MN384256, MN384257, MN384258). In the phylogenetic analysis, we used 32 nucleotide sequences, including 22 Nigerian 18S rDNA sequences and 10 references (Fig. 2).

## Results

In the nPCR targeting the 18S rDNA gene, 33.3% (110/300) of samples were positive. To determine the specific species of organisms infecting horses, individual nPCRs and sequencing were performed using the 18S rDNA positive samples. The overall results for species-specific reactions yielded 13.0% (39/300) for *T. equi*, 3.3% (10/300) *B. caballi*, and 2.7% (8/300) *T. haneyi* (Table 3). Double infection with *T. equi* and *B. caballi* occurred in 2.7% (8/300) of horses and triple infection with *T. equi*, *B. caballi*, and *T. haneyi* was detected in 0.6% (2/300) horses. However, not all 18S rDNA positives could be amplified by species-specific nPCR. Interestingly, two individual horses (Thatcher198 and Castora213 RC) were 18S rDNA sequence positive for *B. bovis* and confirmed to be that by specific nPCR (data not shown).

**Table 3:** Result of nPCR targeting species-specific equine piroplasmids in horses from northwestern Nigeria

Area	Total Number of Sampled	Breed	Positive samples (% positive)		
			*T. equi*	*B. caballi*	*T. haneyi*
Kaduna					
• Igabi	80	Argentine polo ponies	10 (12.5%)	-	-
	8	Thoroughbred	5 (62.5%)	-	-
	3	Criollo	2 (66.7%)	-	-
• Zaria	127	West African Barb	7 (5.5%)	4 (3.1%)	5 (3.9%)
	25	West African Barb	3 (12%)	1 (4%)	1 (4%)
Katsina					
• Mai’adua	32	West African Barb	11 (34.4%)	4 (12.5%)	2 (6.25%)
Jigawa					
• Maigatari	25	West African Barb	1 (4%)	1 (4%)	-

In Igabi Local Government Area (LGA) (Kaduna State), 91 horses were sampled and the prevalence for *T. equi* was 18.7% (17/91). *B. caballi* and *T. haneyi* were not detected. In Zaria LGA (Kaduna State), 152 horses were sampled and the nPCR prevalence of *T. equi* was 6.6% (10/151), while *B. caballi* had 3.3% (5/151) and *T. haneyi* was 4.9% (6/151). Double infection with *T. equi* and *B. caballi* was 3.3% (5/151). In Mai’adua LGA (Katsina state), 32 horses were sampled and the nPCR prevalence for *T. equi* was 34.4% (11/32), *B. caballi* 12.5% (4/32), and *T. haneyi* were 6.25% (2/32). Double infection with *T. equi* and *B. caballi* were 6.25% (2/32) and triple infections with *T equi*, *B. caballi*, and *T. haneyi* were 6.25% (2/32). In Maigatari LGA (Jigawa state), 25 horses were sampled and the nPCR result for *T. equi* was 4% (1/25) and *B. caballi* 4% (1/25). Double infection with *T. equi* and *B. caballi* was found in 4% of the samples (1/25).

In this study, we were able to sequence 61 products of 18S-nPCR and 57 were closely related to *Theileria* that infect horses, two were *B. caballi* and two were *B. bovis*. The familiar clade members that were recognized for *Theileria* with the highest percent similarity to the query sequence were 28 as clade A, 13 as clade C, and 16 as clade D. All the familiar clades were found in Nigeria with exception of clades B and E. To construct the phylogenetic tree, we used 22 randomly selected Nigerian samples of which 18 were closely related to *Theileria* spp. that infect horses, two were *B. caballi* and two were *B. bovis* (Fig. 2). For phylogenetic analysis, sequences were manually checked by alignment to verify that no duplicate sequences were used in the phylogram. No unique sequences were left out of the analysis.

The occurrence of *T. equi* clade A was relatively high in the northwestern part of Nigeria. *T. equi* clade A for Zaria was 18, Igabi 3, Mai’adua 5, and Maigatari 2. Clade C for Zaria was 9, Igabi 1, Mai’adua 3, and Maigatari 0. While for clade D, Zaria had 8, Igabi 2, Mai’adua 4, and Maigatari 2.

## Discussion

The emphasis of the current study is to understand genotypic diversity of protozoan parasites that cause EP in Nigeria using a molecular biology approach. In Nigeria, horses are an important commodity for recreation, sport, transportation, and work. Igabi LGA (Kaduna) has one of the highest numbers of polo ponies in the region, both exotic and indigenous, and annually hosts international polo tournaments, horse racing, and other horse-related activities. Zaria LGA hosts the most prestigious traditional horse procession (durbar), as well as polo and horse racing. This region has one of the largest populations of horses in Kaduna state. Hundreds of horse transits in and out of Zaria, particularly the ancient city, are coming mostly from different parts of northern Nigeria and the Niger Republic. A variety of events occur in this region including twice a year horse processions (durbar), polo events, and horse racing. These events have made Zaria an epidemiologically important niche for disease transmission, infection, and enabling possible adaptation of apicomplexans to other hosts, which could potentially result in various clinical manifestations that relate to EP that have not yet been fully recognized.

Though the number of horses sampled in Zaria was higher compared to other LGAs, it was evident that the result in this area represented the genotypes and parasites found in the other areas. Katsina and Jigawa both share a substantial border with the Niger Republic; the markets were part of the network of centuries-old markets when the region played an important role as the hub of trans-Saharan trade routes. These livestock markets linked into the road networks and settlement patterns, which makes it easier to transport animals across Nigeria. The movement of horses across the northern borders of Nigeria from various neighboring countries for various purposes could have been responsible for the pattern of results seen in the study areas. Therefore, these results could be an epidemiological reflection of EP status in West African Sub-region.

*T. equi* genotpes A C and D were identified in Kadunga and Kasinga, while genotypes A and D were only detected in Jigawa*.* Significantly, *T. equi* clades B and E were absent in all the samples analyzed. Consistent with other studies, genotype diversity of *Theileria* was reported in horses in South Africa, Israel, USA, and Spain (Criado-Fornelio et al. 2003; Bhoora et al. 2009; Hall et al. 2013; Bhoora et al. 2019; Coultous et al. 2020). Bhoora et al. (2019) found clades A, B, C, and D, but not E, in a survey of horses and zebras in South Africa. Coultous et al. (2020) having assayed only for clades A–D (but not E), finding clades A, C, and D in Gambia. Similar to our data, clade B sequences were not identified in Gambia, another west African country. In both studies, coinfections with 2 or more clade members were common, especially in the case of clades A and C.

Since there is no restriction on horse importation, the current horse importation strategy in Nigeria could lead to the introduction of these clades in the future. A large number of the Argentine polo ponies were negative for EP. These negative results could be related to the routine health care management practice which involves the administration of imidocarb which could have led to the persistently low parasitemia that usually follows a fluctuating parasite-load pattern in recovered horses (Ueti et al. 2005; Ueti et al. 2008).

Following the serendipitous discovery of *T. haneyi*, there were questions raised on whether co-infection with *T. equi* and *T. haneyi* occurs naturally. The data obtained from this work prove that *T. equi* and *T. haneyi* can co-infect naturally and additionally with *B. caballi*. Experimental co-infection of *T. equi* and *T. haneyi* was reported (Sears et al. 2019). One of the six polo ponies from Argentina that arrived in Igabi LGA had *T. haneyi*. It was evident that *T. haneyi* was not detected at the time of arrival. The failure to detect *T. haneyi* is no surprise; Knowles et al. (2018) reported that *T. haneyi* lacks *ema-1* which allows the parasite to elude the current regulatory cELISA test which is based on EMA-1 of the immunodominant EMA superfamily and a specific monoclonal antibody (Knowles et al. 2018). The 18S-rDNA nested PCR with sequencing detected larger numbers of *T. haneyi* as compared to the species-specific-nested PCR. This discrepancy is potentially associated with a reduced sensitivity to the gene coding for a protein of unknown function used to detect *T. haneyi*. The *T. haneyi* reference JQ390047 in Fig. 2 branches off earlier than clade C identified from Nigeria. The identity of the Nigerian clade C samples to references EU888903 and JQ390047 are 100% and 99.53%, respectively. The *T. haneyi* reference JQ390047 has only 2 nucleotide differences with the Nigerian clade C samples yet gives the appearance of greater distinctness in the phylogram. For comparison, *T. equi* clade A reference JX177671 has 24 nucleotide differences compared to clade C members in the phylogram, demonstrating the distinction of a separated clade. No samples reported here were 100% identical to *T. haneyi*.

Compared with equine theileriosis, *B. caballi* is not predominant in areas that are EP-prone as confirmed using molecular assays. The occurrence of *B. caballi* ranges from 0 to 19.3% depending on the area (Heim et al. 2007; Motloang et al. 2008; Mahmoud et al. 2016; Sumbria et al. 2016). However, a high prevalence of *B. caballi* was reported in Mongolia (Munkhjargal et al. 2013; Mans et al. 2015) and Italy (Laus et al. 2013). The low number of *B. caballi* detected in this study could be a result of the very low parasitemia that characterizes *B. caballi* infections, which rarely exceeds 1% and the removal of *B. caballi* over a period of time by the horse immune system (Hanafusa et al. 1998; Guclu and Karaer 2007).

An interesting finding was the detection of *B. bovis* DNA from horses that arrived in Nigeria from Argentina. Two animals were both 18S rDNA-PCR positive with sequences closely related to *B. bovis*, and those samples were also *B. bovis* species-specific nPCR positive. Herein, we have presented that imported horses could be infected with *B. bovis*, where different strains could be distributed to various areas of the world, considering the human-driven global horse movement. Whether *B. bovis* established infection in these horses is unknown, further investigation is needed to rule out the possibility of *B. bovis*, and other bovine Apicomplexan parasites, to establish infections in horses. Nonetheless, if *B. bovis* established infection, these horses could be sources of parasite dissemination. This could necessitate testing horses frequently whenever they are moving into disease-controlled areas. This is supported by the recent identification of *T. annulata*, *B. ovis*, and *B. canis* species in horse blood samples in Turkey (Ozubek and Aktas 2018), and a more recent detection of *T. equi* and *T. velifera* in guard dogs kept in horse stables in Saudi Arabia (Salim et al. 2019). Also, *T. equi* has been detected in clinically ill dogs in Croatia (Beck et al. 2009) and South Africa (Matjila et al. 2008). This seeming lack of host specificity has raised a question on the clinical implications and impact of atypical piroplasmosis (Ozubek and Aktas 2018). Equid and non-equids species are likely to share closely related parasites that could be transmitted by a wide range of tick vectors. With the seeming emergence of atypical piroplasmosis in a variety of species worldwide, there will be a need to elucidate the tick vector, parasite, and host relationships that lead to the development of clinical disease.

## Conclusion

It is evident that *T. equi*, *T. haneyi*, and *B. caballi* are present in Nigeria and circulating on an endemic basis among the equine species supported by the current study. The presence of infected horses may play a critical role in the spread of these pathogens via tick vectors. However, it is unknown which ticks are vector competent in Nigeria. Determination of the various competent tick vectors responsible for parasite transmission warrants additional study. Therefore, surveillance and restriction of the international movement of equids are required to prevent the introduction of infected horses into Nigeria to avoid pathogen dissemination.
